# Das Geschlechterparadoxon in der gesundheitlich beeinträchtigten Lebenszeit – Ende eines Mythos?

**DOI:** 10.1007/s00103-024-03877-7

**Published:** 2024-04-25

**Authors:** Marc Luy

**Affiliations:** Vienna Institute of Demography, Österreichische Akademie der Wissenschaften, Dominikanerbastei 16, 1010 Wien, Österreich

**Keywords:** Lebenserwartung, Gesundheit, Geschlechterunterschied, Mortalitätseffekt, Differential Item Functioning, Life expectancy, Health, Gender difference, Mortality effect, Differential item functioning

## Abstract

**Hintergrund:**

Frauen leben länger als Männer, verbringen aber mehr Lebensjahre mit gesundheitlicher Beeinträchtigung. In diesem Beitrag wird untersucht, inwieweit dieses Geschlechterparadoxon durch 2 Faktoren erklärt werden kann: den „Mortalitätseffekt“, der aus der höheren Lebenserwartung der Frauen resultiert, und das „Differential Item Functioning“ (DIF), das Geschlechterunterschiede im Berichtsverhalten bezeichnet.

**Methoden:**

Die beeinträchtigte Lebenserwartung im Alter 50 für die Gesundheitsindikatoren Allgemeingesundheit, Einschränkungen und chronische Morbidität wird mit der Sullivan-Methode berechnet. Daten zur Gesundheitsprävalenz stammen aus dem Survey „Gesundheit in Deutschland aktuell“ (GEDA) des Jahres 2012, Daten zur Mortalität aus der „Human Mortality Database“. Die Geschlechterdifferenz in der beeinträchtigten Lebenserwartung wird mittels Dekomposition in den Mortalitäts- und den Gesundheitseffekt zerlegt. Letzterer wird schließlich auf der Grundlage von Vignetten aus der ersten Welle des „Survey of Health, Ageing and Retirement in Europe“ (SHARE) um DIF-Effekte bereinigt.

**Ergebnisse:**

Das Geschlechterparadoxon lässt sich für alle 3 betrachteten Gesundheitsindikatoren nicht nur teilweise, sondern vollständig durch Mortalitätseffekt und DIF auflösen. Nach Berücksichtigung dieser beiden Faktoren kehrt sich die Geschlechterdifferenz in der beeinträchtigten Lebenserwartung von höheren Werten für Frauen in höhere Werte für Männer um.

**Diskussion:**

Die Ursachen für das Geschlechterparadoxon sind sehr komplex und die Frauen-Männer-Differenzen in gesamter und beeinträchtigter Lebenserwartung gehen nicht unbedingt in widersprüchliche Richtungen. Das Ausmaß der höheren beeinträchtigten Lebenserwartung der Frauen hängt entscheidend vom zugrunde liegenden Gesundheitsindikator ab und wird zum größten Teil durch den Mortalitätseffekt erklärt.

## Abstract (en)

**Background:**

Women live longer than men, but they spend more life years with health-impairment. This article examines the extent to which this gender paradox can be explained by two factors: the “mortality effect,” which results from the higher life expectancy of women, and “differential item functioning” (DIF), which refers to gender differences in reporting behavior.

**Methods:**

Impaired life expectancy at age 50 is calculated for the health indicators general health, limitations, and chronic morbidity using the Sullivan method. Data on health prevalence are obtained from the 2012 survey “Gesundheit in Deutschland aktuell” (GEDA), data on mortality is taken from the Human Mortality Database. The gender difference in impaired life expectancy is decomposed into the mortality effect and the health effect. The latter is finally adjusted for DIF effects on the basis of vignettes from the 2004 SHARE survey.

**Results:**

The gender paradox can be resolved not only partially but completely for all three health indicators considered by the mortality effect and DIF. After taking these two factors into account, the gender difference in impaired life expectancy reverses from higher values for women to higher values for men.

**Discussion:**

The causes of the gender paradox are highly complex and the differences between women and men in total and impaired life expectancy are not necessarily going into contradictory directions. The extent of women’s higher impaired life expectancy depends decisively on the underlying health indicator and is largely explained by the mortality effect.

## Hintergrund

Beginnend mit den 1920er-Jahren hat sich eine Vorstellung über die Geschlechterdifferenzen in Gesundheit und Mortalität etabliert, die von Lorber und Moore in dem einprägsamen Satz: „Women get sicker, but men die quicker“, zusammengefasst wurde [1, S. 13]. Tatsächlich erscheinen vor dem Hintergrund der höheren Lebenserwartung der Frauen die Studienergebnisse zu den Geschlechterdifferenzen in der Morbidität überraschend, wonach Frauen im Durchschnitt einen schlechteren Gesundheitszustand aufweisen als Männer [2, 3] und sie mehr Lebensjahre sowie einen größeren Teil der gesamten Lebenszeit mit beeinträchtigter Gesundheit verbringen [4, 5]. Selbst bei Ausschluss von Gesundheitsbeeinträchtigungen, die mit der Reproduktion in Verbindung stehen, bleibt ein erheblicher Geschlechterunterschied bei akuten Erkrankungen und kurzfristigen Einschränkungen bestehen [6]. Außerdem nimmt die körperliche Funktionalität bei Frauen schneller ab und sie zeigen eine geringere Wahrscheinlichkeit, sich von starken gesundheitlichen Einschränkungen zu erholen [3]. Darüber hinaus wurde gezeigt, dass Frauen mehr Gesundheitsdienste in Anspruch nehmen [7, 8] und im Allgemeinen mehr Medikamente verwenden als Männer [9]. Diese Widersprüche zu den Sterblichkeitsunterschieden zwischen den Geschlechtern haben in der englischsprachigen Literatur zu zahlreichen Veröffentlichungen geführt, die das Phänomen mit Begriffen wie „Gender and Health Paradox“ [10], „Morbidity Paradox“ [11], „Morbidity-Mortality Paradox“ [12], „Male-Female Health-Survival Paradox“ [13, 14] oder „Male-Female Health-Mortality Paradox“ [15] beschreiben.

In jüngerer Zeit wurde die Existenz eines generellen und auf alle Gesundheitsaspekte verallgemeinerbaren Paradoxons jedoch zunehmend infrage gestellt. Verschiedene Studien haben gezeigt, dass die gesundheitlichen Unterschiede zwischen Frauen und Männern mit Alter, Gesundheitsindikator, Kalenderzeit und sozialem Kontext variieren (siehe z. B. [10, 11, 16–18]). Daneben ist zu berücksichtigen, dass die meisten Erkenntnisse über Geschlechterdifferenzen in der Morbidität auf Studien beruhen, die sich auf breite Gesundheitskategorien konzentrieren wie Allgemeingesundheit, Einschränkungen im Alltagsleben oder chronische Krankheiten, während die Analyse spezifischer gesundheitlicher Defizite zu sehr unterschiedlichen Ergebnissen führen kann [12]. Nichtsdestotrotz existiert die Idee eines generell vorherrschenden „Geschlechterparadoxons“ auch heute noch.

Aus der Fülle an unterschiedlichsten Aspekten bezüglich der Geschlechterdifferenzen in der Gesundheit greift der vorliegende Beitrag jenen der Lebensjahre heraus. Dieser steht im direkten Zusammenhang mit einem der zentralen Gedanken des Geschlechterparadoxons, nämlich dass Frauen trotz der geringeren Sterblichkeit mehr Lebensjahre in beeinträchtigter Gesundheit verbringen als Männer. Dies kann mit dem demographischen Indikator „gesunde Lebenserwartung“ gezeigt werden, in dem die über alle Alter vorherrschende Sterblichkeit und Gesundheit in einer für verschiedene Bevölkerungen vergleichbaren Maßzahl zusammengefasst werden [19]. Hierfür wird in der Regel auf die bereits erwähnten breiten Gesundheitskategorien zurückgegriffen, die in vielen repräsentativen Surveys erhoben werden. Bezüglich der durch diesen Indikator abgebildeten Geschlechterdifferenzen stellten Luy und Minagawa [20] die Hypothese auf, dass die für Frauen im Vergleich zu den Männern höhere Anzahl an mit gesundheitlichen Beeinträchtigungen verbrachten Lebensjahren vor allem aus ihrer höheren Lebenserwartung resultiert. Unterstützt wurde die Hypothese in dieser Arbeit durch eine für 187 Länder durchgeführte Analyse der Korrelation zwischen den Geschlechterdifferenzen in der Lebenserwartung und der gesunden Lebenserwartung nach der „Global Burden of Disease Study“ des Jahres 2010, der ein globaler Gesundheitsindikator zugrunde liegt, welcher abgeleitet wurde aus dem Gesundheitsverlust durch 291 Krankheiten und Verletzungen, 1160 Folgeerkrankungen dieser Krankheiten und Verletzungen sowie 67 Risikofaktoren oder Risikofaktorenkombinationen [21].

In dem vorliegenden Artikel wird die Hypothese von Luy und Minagawa [20] für die deutsche Bevölkerung getestet, wobei der in der Hypothese beschriebene „Mortalitätseffekt“ (ME) – der besagt, dass die höhere Lebenserwartung der Frauen unmittelbar zu einer höheren Anzahl an mit gesundheitlichen Beeinträchtigungen verbrachten Lebensjahren im Vergleich zu den Männern führt – hier durch die Betrachtung von 3 unterschiedlichen Gesundheitsindikatoren als Basis für die gesunde Lebenserwartung untersucht wird. Damit stellt die vorliegende Untersuchung die Fortführung einer jüngst von Luy [22] durchgeführten Studie dar, in der für eine Vielzahl von Subpopulationen der deutschen Bevölkerung der Zusammenhang zwischen Lebenserwartung und mit Gesundheitsproblemen verbrachten Lebensjahren analysiert wurde. Dabei zeigte sich sowohl bei den Frauen als auch bei den Männern ein stark positiver Zusammenhang: je höher die Lebenserwartung, desto höher die Anzahl der beeinträchtigten Lebensjahre. Um die Übertragung dieser Erkenntnisse auf die Geschlechterdifferenzen zu testen, werden in dieser Arbeit die Unterschiede zwischen Frauen und Männern in den beeinträchtigten Lebensjahren durch eine Dekompositionsanalyse zerlegt in den ME und die verbleibenden Geschlechterdifferenzen in Gesundheit, den sogenannten „Gesundheitseffekt“. Auf diese Weise lässt sich schätzen, wie groß die allein auf Gesundheitsunterschiede zurückzuführenden Geschlechterdifferenzen in der beeinträchtigten Lebenserwartung sind.

Die Analyse wird schließlich erweitert durch die zusätzliche Berücksichtigung eines zweiten Effekts, der die Geschlechterdifferenzen in den gesunden bzw. beeinträchtigten Lebensjahren beeinflussen könnte, das sogenannte „Differential Item Functioning“ (DIF). Damit werden Unterschiede im Berichtsverhalten zwischen Bevölkerungen oder Subpopulationen beschrieben, die einen Vergleich von selbstberichteten Informationen verzerren können, wie sie in Surveys erhoben werden. Bezüglich der Geschlechterdifferenzen in der selbst eingeschätzten Gesundheit könnte eine derartige Verzerrung dadurch entstehen, dass Frauen empfindlicher auf körperliche Beschwerden reagieren und eher bereit sind, in Gesundheitsbefragungen Symptome von Beschwerden und Krankheiten anzugeben [23]. Zum Test des DIF-Effekts werden in diesem Artikel sogenannte Vignetten verwendet, mit denen sich die Heterogenität im gruppenspezifischen Berichtsverhalten kontrollieren lässt. Dieser Ansatz wurde bereits zur Identifikation von DIF in verschiedenen Bereichen eingesetzt, z. B. bei der Analyse von Schülerleistungen [24], Lebenszufriedenheit [25], Arbeitszufriedenheit [26] und wirtschaftlichem Wohlstand [27]. Jüngst haben Luy et al. [28] eine Methode vorgestellt, bei der Vignetten verwendet werden, um die Auswirkungen von DIF auf internationale Unterschiede bei den gesunden Lebensjahren auszugleichen. Im Folgenden wird dieses Verfahren verwendet, um den geschätzten Gesundheitseffekt bei den Geschlechterdifferenzen in den gesundheitlich beeinträchtigten Lebensjahren um den DIF-Effekt zu bereinigen.

## Methoden

Die beeinträchtigte Lebenserwartung im Alter 50, BLE(50), für Frauen und Männer wurde mit der von Sullivan [29] entwickelten Methode für die 3 Gesundheitsindikatoren des Minimum European Health Module (MEHM) geschätzt: Allgemeingesundheit („self-perceived health“, SPH), gesundheitsbedingte Einschränkungen im Alltagsleben („limitations“, LIMIT) und das Leben mit einer oder mehreren chronischen Krankheiten (CHRON). Als Datengrundlage diente der Survey aus der Studie „Gesundheit in Deutschland aktuell“ (GEDA) des Robert Koch-Instituts aus dem Jahr 2012 [30], der 10.744 Personen in der Altersgruppe 50 und älter umfasste (53,64 % Frauen; 46,36 % Männer). Damit basiert die vorliegende Untersuchung auf denselben Daten, die auch für die Studie von Luy [22] herangezogen wurde, die mit der hier präsentierten Analyse fortgeführt werden soll. SPH ist der von den Befragten selbst eingeschätzte generelle Gesundheitszustand und wurde in GEDA-2012 erfasst durch die Frage: „Wie ist Ihr Gesundheitszustand im Allgemeinen?“, mit den 5 Antwortmöglichkeiten: „sehr gut“, „gut“, „mittelmäßig“, „schlecht“ und „sehr schlecht“. LIMIT wurde durch die Frage: „In welchem Ausmaß sind Sie durch Krankheit in der Ausübung Ihrer alltäglichen Tätigkeiten dauerhaft eingeschränkt?“, abgefragt mit der Erläuterung: „Wir meinen damit seit mindestens einem halben Jahr“. Zu dieser Frage gab es die 3 Antwortmöglichkeiten: „erheblich eingeschränkt“, „eingeschränkt, aber nicht erheblich“ und „nicht eingeschränkt“. CHRON ist definiert als das Vorhandensein von chronischen Gesundheitsproblemen und basiert auf der Frage: „Haben Sie eine oder mehrere lang andauernde, chronische Krankheiten?“, mit dem erklärenden Hinweis: „Chronische Krankheiten sind lang andauernde Erkrankungen, die ständiger Behandlung und Kontrolle bedürfen, z. B. Diabetes oder Herzerkrankungen“, und den beiden Antwortmöglichkeiten: „Ja“ und „Nein“. Bei allen Fragen gab es auch die Optionen „weiß nicht“ und „keine Antwort“. Für die Schätzung der BLE nach den 3 MEHM-Varianten wurde beeinträchtigte Gesundheit definiert als schlechte oder sehr schlechte Allgemeingesundheit, erhebliche Einschränkungen bei Alltagstätigkeiten und das Vorhandensein chronischer Gesundheitsprobleme.

**Figure 1:**
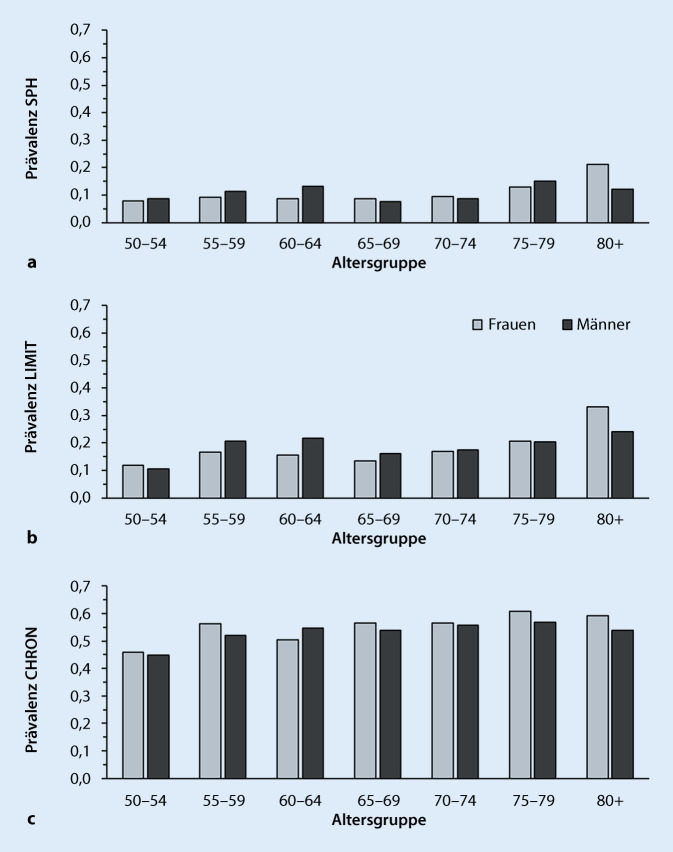

Abb. 1 zeigt die Prävalenzanteile beeinträchtigter Gesundheit für die 3 MEHM-Gesundheitsindikatoren für die Altersgruppen 50–54, 55–59, …, 80+, getrennt für Frauen und Männer nach den GEDA-2012-Daten. Die für die Bestimmung der BLE(50) erforderlichen Sterbetafeln wurden auf Basis der Sterbewahrscheinlichkeiten des Jahres 2012 für Frauen und Männer für alle Einzelalter von Alter 50 bis 110 mit den Daten der „Human Mortality Database“ (HMD) berechnet [31]. Die mit beeinträchtigter Gesundheit verbrachten Lebensjahre resultieren aus Multiplikation der aus diesen Sterbetafeln ermittelten insgesamt gelebten Jahre in den Altersgruppen 50–54, 50–59, …, 80+ mit den jeweiligen Prävalenzanteilen. Den Startpunkt der Analysen bilden Unterschiede in der BLE(50) zwischen Frauen und Männern. Diese Differenzen wurden im nächsten Schritt mit der Dekompositionsmethode von Nusselder und Looman [32] zerlegt in den Mortalitätseffekt (ME) und den Gesundheitseffekt (GE). Schließlich wurde der GE dem unterschiedlichen Berichtsverhalten von Frauen und Männern angepasst. Hierfür wurden die altersspezifischen Prävalenzen für SPH, LIMIT und CHRON der Frauen um den DIF-Effekt bereinigt. Dies geschah auf Basis von Gesundheitsvignetten, die in einer Unterstichprobe der ersten Welle des „Survey of Health, Ageing and Retirement in Europe“ (SHARE) enthalten waren [33].^1^ Für Deutschland umfasste das SHARE-2004-Subsample mit den Vignetten 508 Personen.

Vignetten sind kurze Beschreibungen fiktiver Personen, die bestimmte Gesundheitsprobleme in mehr oder weniger starkem Ausmaß aufweisen [34]. Beispiele aus SHARE-2004 sind: „Lukas hat einmal im Monat Kopfschmerzen, die nach der Einnahme einer Tablette aufhören. Während der Kopfschmerzen kann er sich weiter um seine alltäglichen Aufgaben kümmern“ oder „Hans hat Schmerzen, die während der Arbeit bis in den rechten Arm und das Handgelenk hinunterziehen. Abends geht es ihm etwas besser, wenn er nicht mehr an seinem Computer arbeitet“.^2^ Die Survey-Teilnehmerinnen und -Teilnehmer wurden dann gebeten, den Schweregrad der beschriebenen Gesundheitsprobleme zu bewerten, wobei die Antwortkategorien die 5 Möglichkeiten „keine“, „wenige“, „mäßige“, „große“ und „extreme“ umfassten. Die erste SHARE-Erhebung beinhaltete insgesamt 27 Gesundheitsvignetten, die 7 unterschiedliche Gesundheitsmerkmale beschreiben: jeweils 3 Vignetten zu den Merkmalen „körperliche Beschwerden oder Schmerzen“ (zu denen o. g. Beispiele gehören), „Schlafstörungen“, „Schwierigkeiten, sich zu bewegen“, „Probleme, sich zu konzentrieren oder sich an Dinge zu erinnern“, „Kurzatmigkeit“, „sich traurig, bedrückt oder deprimiert fühlen“ und 9 Vignetten zu „Einschränkungen, durch die man bei der Art oder im Umfang der Arbeit, die man ausüben kann, behindert wird“. Zusätzlich zu der Einschätzung des Gesundheitszustands der Vignettencharaktere wurden die Befragten gebeten, ihre eigene Gesundheit für die gleichen 7 Gesundheitsmerkmale anhand derselben Antwortskala zu bewerten.

Eine DIF-Bereinigung der Prävalenzwerte der MEHM-Gesundheitsindikatoren würde eigentlich spezielle Vignetten für SPH, LIMIT und CHRON erfordern. Dies ist jedoch aufgrund der sehr allgemein gehaltenen Fragen, wie sie im GEDA-Survey zu finden sind, nicht möglich. Selbst bezüglich des Gesundheitsindikators LIMIT, der durch die 9 Vignetten zu Einschränkungen in Art und Umfang der Arbeit der entsprechenden GEDA-Frage vergleichbar erfasst ist, unterscheiden sich die Fragestellungen in ihrer Präzision. Deshalb wurde in dieser Studie der von Luy et al. [28] entwickelte Ansatz angewandt, auf Basis der Vignettenfragen für die eigene Gesundheit der Befragten diejenigen Gesundheitsmerkmale zu bestimmen, die statistisch signifikant mit den 3 Gesundheitsindikatoren SPH, LIMIT und CHRON in Zusammenhang stehen. Aufgrund der kleinen Stichprobengröße des deutschen Subsamples der ersten SHARE-Welle wurde das Verfahren von Luy et al. [28] hier leicht abgeändert und nur für die gesamte Stichprobe getrennt für Frauen und Männer, aber ohne Altersdifferenzierung angewandt.

Als Maßstab für die DIF-Korrektur wurden für alle Vignettengesundheitsmerkmale die Ausprägungen „große Probleme“ und „extreme Probleme“ gewählt. Durch die Reduktion auf die schweren Symptome sollte eine Überschätzung des DIF-Effekts verhindert werden. Für die gewählten Definitionen von beeinträchtigter Gesundheit in den 3 MEHM-Indikatoren sollte das durch diese Reduktion beschriebene Ausmaß an Gesundheitsproblemen bei SPH (schlechte oder sehr schlechte Allgemeingesundheit) und LIMIT (erhebliche Einschränkungen) gut übereinstimmen. Bei CHRON hingegen, bei dem allein das Vorhandensein chronischer Gesundheitsprobleme einen schlechten Gesundheitszustand definiert, führt die Reduktion auf große und extreme Probleme vermutlich zu einer engeren Definition von gesundheitlicher Beeinträchtigung.

Die Anteile der von Frauen und Männern eingeschätzten Vignettencharaktere mit großen und extremen Problemen deuten tatsächlich auf die Existenz von Geschlechterdifferenzen in der Einschätzung identischer Gesundheitsmerkmale hin (Tab. 1, Spalte F/M Ratio). Bei 5 der 7 Gesundheitsmerkmale weisen Frauen mehr Vignettencharakteren eine schlechte Gesundheit zu als Männer, nämlich bei körperlichen Beschwerden oder Schmerzen, Schlafstörungen, Schwierigkeiten, sich zu bewegen, Traurigkeit, Bedrückung oder Depression und Einschränkungen in Art oder Umfang der Arbeit. Männer weisen hingegen bei den beiden Gesundheitsmerkmalen Probleme mit Konzentration oder Erinnerung und Kurzatmigkeit mehr Vignetten große oder extreme Gesundheitsprobleme zu.

**Table 1:** 

		*p*-Werte logistische Regression		
Gesundheitsmerkmal	F/M Ratio	SPH	LIMIT	CHRON
Körperliche Beschwerden oder Schmerzen	1,11	*0,001*	0,060	*0,042*
Schlafstörungen	1,06	*0,031*	0,630	0,158
Schwierigkeiten, sich zu bewegen	1,02	0,572	*0,000*	*0,042*
Probleme mit Konzentration oder Erinnerung	0,96	0,127	0,439	0,675
Kurzatmigkeit	0,95	0,066	0,171	0,228
Traurigkeit, Bedrückung oder Depression	1,05	*0,031*	0,080	0,165
Einschränkungen in Art oder Umfang der Arbeit	1,06	*0,000*	*0,000*	*0,006*
DIF-Korrekturfaktor	–	1,065	1,048	1,059

Anmerkungen: Alle Variablen sind als Dummy-Variablen kodiert mit dem Wert 1 für das Vorherrschen von Gesundheitsproblemen (für die jeweilige Definition siehe Text); logistische Regressionsanalyse mit Forward Stepwise Selection Models (LR) mit *p* < 0,05 als Grenzwert (unter dem Grenzwert liegende *p*-Werte sind kursiv hervorgehoben); Daten: SHARE-*Deutschland 2004* (Vignetten Unterstichprobe)

*MEHM* Minimum European Health Module, *DIF* Differential Item Functioning, *SPH* Allgemeingesundheit, *LIMIT* Einschränkungen, *CHRON* chronische Krankheit(en)

Für die Bestimmung der DIF-Korrekturfaktoren für die 3 MEHM-Gesundheitsindikatoren wurden dann in einem ersten Schritt die durch die Vignetten beschriebenen Gesundheitsmerkmale identifiziert, die bei den Teilnehmerinnen und Teilnehmern der SHARE-2004-Erhebung mit einer gesundheitlichen Beeinträchtigung in den 3 MEHM-Indikatoren SPH, LIMIT und CHRON in Verbindung stehen. Für die Auswahl wurden auf Basis der von den SHARE-Befragten für sich selbst berichteten Gesundheit für jeden MEHM-Indikator getrennt logistische Regressionsanalysen (Forward Stepwise) durchgeführt. Dabei wurden die MEHM-Indikatoren als abhängige und die Vignettengesundheitsmerkmale als unabhängige Variablen ohne zusätzliche Kontrollvariablen eingeschlossen. Als Einschlusskriterium galt ein Signifikanzniveau von *p* < 0,05.^3^ Die aus diesen Analysen resultierenden *p*-Werte sind in Tab. 1 zu finden, wobei die *p*-Werte der für die Bestimmung der DIF-Korrekturfaktoren ausgewählten Gesundheitsmerkmale kursiv hervorgehoben sind. Bei Beeinträchtigung im Indikator SPH sind dies körperliche Beschwerden oder Schmerzen, Schlafstörungen, Gefühl von Traurigkeit, Bedrückung oder Depression und Einschränkungen in Art oder Umfang der Arbeit. Beeinträchtigungen im Gesundheitsindikator LIMIT stehen mit Schwierigkeiten, sich zu bewegen, und Einschränkungen in Art oder Umfang der Arbeit in Verbindung. Das Vorhandensein chronischer Gesundheitsprobleme (CHRON) steht in statistisch signifikantem Zusammenhang mit körperlichen Beschwerden oder Schmerzen, Schwierigkeiten, sich zu bewegen, und Einschränkungen in Art oder Umfang der Arbeit.

Für den nächsten Schritt wurden die Vignetten zu den für die jeweiligen MEHM-Indikatoren relevanten Gesundheitsmerkmalen herangezogen. Unter diesen wurde dann die Anzahl an Vignetten ermittelt, denen von den Frauen und den Männern des SHARE-2004-Samples große oder extreme Gesundheitsprobleme zugewiesen wurden. Aus diesen wurden Frauen-Männer-Quotienten der mit Gesundheitsproblemen eingeschätzten Vignetten berechnet, die schließlich als DIF-Korrekturfaktoren für die Prävalenz von Gesundheitsproblemen in den 3 MEHM-Gesundheitsindikatoren verwendet wurden (siehe unterste Zeile in Tab. 1). Hierfür wurden die SPH-, LIMIT- und CHRON-Prävalenzwerte der Frauen in jeder Altersgruppe durch den jeweiligen Korrekturfaktor dividiert. Aus diesen DIF-bereinigten Prävalenzwerten wurde schließlich die DIF-bereinigte beeinträchtigte Lebenserwartung (BLE*) für Frauen berechnet und deren Differenz zu den BLE-Werten (nicht bereinigte beeinträchtigte Lebenserwartung) für die Männer bestimmt. Diese wurde im letzten Schritt mit dem oben beschriebenen Dekompositionsverfahren in DIF-bereinigte Mortalitäts- und Gesundheitseffekte zerlegt, um so eine um Mortalitätseffekt und DIF bereinigte Geschlechterdifferenz in den beeinträchtigten Lebensjahren zu schätzen. Diese werden im Folgenden als ME* und GE* gekennzeichnet.

## Ergebnisse

Tab. 2 zeigt die Schätzungen für die Lebenserwartung und die beeinträchtigte Lebenserwartung im Alter 50 auf Basis der 3 Gesundheitsindikatoren des MEHM für Frauen und Männer sowie die jeweiligen Geschlechterunterschiede als Frauen-Männer-Differenz. Demnach hatten Frauen im Referenzjahr 2012 eine um 4,79 Jahre höhere Lebenserwartung im Alter 50. Auch die gesundheitlich beeinträchtigten Lebensjahre sind bei den Frauen bei jedem Gesundheitsindikator höher, wie es der Begriff des Geschlechterparadoxons zum Ausdruck bringt. Allerdings unterscheidet sich das Ausmaß der bei den Frauen höheren Anzahl an ungesunden Lebensjahren zwischen den zugrunde liegenden Gesundheitsindikatoren. Auf Basis der Allgemeingesundheit (SPH) ist die Geschlechterdifferenz in der beeinträchtigten Lebenserwartung mit 0,82 Jahren am geringsten. Am ausgeprägtesten ist der gesundheitliche Unterschied bei den chronischen Krankheiten (CHRON). Hier verbringen Frauen 3,32 Lebensjahre mehr als Männer mit gesundheitlichen Beeinträchtigungen. Bei den gesundheitlich bedingten Einschränkungen in Alltagstätigkeiten (LIMIT) liegt die Geschlechterdifferenz mit 1,10 Jahren dazwischen.

**Table 2:** 

		BLE(50)		
Population	LE(50)	SPH	LIMIT	CHRON
Frauen	34,24	4,00	6,55	18,86
Männer	29,45	3,19	5,46	15,53
Differenz (Frauen-Männer)	4,79	0,82	1,10	3,32

Anmerkung: Abweichungen zwischen den angegebenen Summen bzw. Differenzen sind möglich wegen Rundungseffekten; Daten: GEDA 2012

*SPH* Allgemeingesundheit, *LIMIT* Einschränkungen, *CHRON* chronische Krankheit(en)

Bei Betrachtung der Zahlen in Tab. 2 fällt auf, dass das Ausmaß der Geschlechterdifferenzen in den beeinträchtigten Lebensjahren mit der Höhe der mit den jeweiligen Gesundheitsbeeinträchtigungen verbrachten Lebensjahre korreliert. Diese sind bei den chronischen Krankheiten mit 18,86 Jahren bei den Frauen und 15,53 Jahren bei den Männern deutlich höher als bei den anderen beiden Gesundheitsindikatoren. Mit schlechter oder sehr schlechter Allgemeingesundheit beträgt die beeinträchtigte Lebenserwartung 4,00 Jahre bei den Frauen und 3,19 Jahre bei den Männern, bei den erheblich eingeschränkten Alltagstätigkeiten liegen die Werte bei 6,55 Jahren für Frauen und 5,46 Jahren für Männer. Es zeigt sich hier also, dass das Ausmaß des Geschlechterparadoxons umso ausgeprägter ist, je größer die Gesamtzahl an mit gesundheitlichen Beeinträchtigungen verbrachten Lebensjahren ist. Diese Beobachtung führt unmittelbar zur Hypothese von Luy und Minagawa [20], die besagt, dass die bei Frauen höhere Anzahl an ungesunden Lebensjahren eine unmittelbare Folge ihrer insgesamt höheren Lebenserwartung ist.

Zum Test dieser Hypothese wurden die Geschlechterdifferenzen in der beeinträchtigten Lebenserwartung im Alter 50 durch Dekomposition in den Mortalitätseffekt (ME) und den Gesundheitseffekt (GE) zerlegt. Letzterer stellt die Geschlechterdifferenz in den beeinträchtigten Lebensjahren dar, die allein auf die Geschlechterunterschiede in der Gesundheit zurückzuführen sind, also bereinigt um den Effekt der unterschiedlichen Gesamtzahl an Lebensjahren von Frauen und Männern. Die in Tab. 3 präsentierten Ergebnisse der Dekompositionsanalyse zeigen, dass der Großteil der höheren beeinträchtigten Lebenserwartung der Frauen tatsächlich auf den Mortalitätseffekt zurückzuführen ist. Bei der Allgemeingesundheit reduziert die ME-Bereinigung der beeinträchtigten Lebenserwartung, wie sie durch den GE dargestellt wird, die von den Frauen höhere Anzahl an mit Beeinträchtigungen verbrachten Lebensjahren von 0,82 auf 0,15 Jahre. Bei den Einschränkungen im Alltagsleben ändert sich die Geschlechterdifferenz von einer ursprünglich bei den Frauen um 1,10 Jahre höheren beeinträchtigten Lebenserwartung nach Bereinigung um den ME sogar in einen um 0,03 Jahre geringeren Wert. Die größte Geschlechterdifferenz ist auch im Gesundheitseffekt bei den mit chronischen Krankheiten verbrachten Lebensjahren zu finden. Allerdings verringert die ME-Bereinigung die diesbezügliche Frauen-Männer-Differenz von 3,32 auf 0,62 Jahre.

**Table 3:** 

		Dekomposition Diff. BLE(50)	
Gesundheitsindikator	Diff. BLE(50)	ME	GE
Allgemeingesundheit (SPH)	0,82	+0,67	+0,15
Einschränkungen (LIMIT)	1,10	+1,13	−0,03
Chronische Krankheit (CHRON)	3,32	+2,70	+0,62

Anmerkung: Abweichungen zwischen den angegebenen Summen bzw. Differenzen sind möglich wegen Rundungseffekten; Daten: GEDA 2012

Tab. 4 zeigt schließlich die Ergebnisse der durchgeführten Analysen für die zusätzliche Berücksichtigung des unterschiedlichen Berichtsverhaltens von Frauen und Männern. In der ersten Spalte sind die DIF-bereinigten Werte für die beeinträchtigte Lebenserwartung, BLE(50)*, für Frauen für die 3 MEHM-Gesundheitsindikatoren zu finden. Da die DIF-Bereinigung zu einer Reduktion der Prävalenzwerte für beeinträchtigte Gesundheit bei den Frauen führt, liegen die BLE(50)*-Werte unterhalb der ursprünglichen in Tab. 3 zu findenden Werte für die unbereinigte BLE(50). Die daneben stehende Spalte zeigt die Differenzen zu den BLE(50)-Werten für Männer, also die DIF-bereinigte Frauen-Männer-Differenz in den gesundheitlich beeinträchtigten Lebensjahren. Diese sind folgerichtig ebenfalls geringer als die ursprünglichen Geschlechterdifferenzen in der beeinträchtigten Lebenserwartung (Tab. 3). Schließlich zeigen die beiden letzten Spalten in Tab. 4 die Ergebnisse der Dekompositionsanalyse, mit der die Frauen-Männer-Differenz in BLE(50)* in die DIF-bereinigten Mortalitäts- und Gesundheitseffekte ME* und GE* zerlegt wurde. Letztere zeigen für alle MEHM-Indikatoren negative Werte, also eine geringere Anzahl an gesundheitlich beeinträchtigten Lebensjahren bei den Frauen.

**Table 4:** 

			Dekomp. Diff. BLE(50)*	
Gesundheitsindikator	BLE(50)F*	Diff. BLE(50)*	ME*	GE*
Allgemeingesundheit (SPH)	3,76	0,57	+0,64	−0,07
Einschränkungen (LIMIT)	6,25	0,79	+1,10	−0,31
Chronische Krankheit (CHRON)	17,81	2,28	+2,62	−0,34

Anmerkung: Abweichungen zwischen den angegebenen Summen bzw. Differenzen sind möglich wegen Rundungseffekten, BLE(50)F* = DIF-bereinigte BLE für Frauen; Daten: GEDA 2012, SHARE 2004

*DIF* Differential Item Functioning

**Figure 2:**
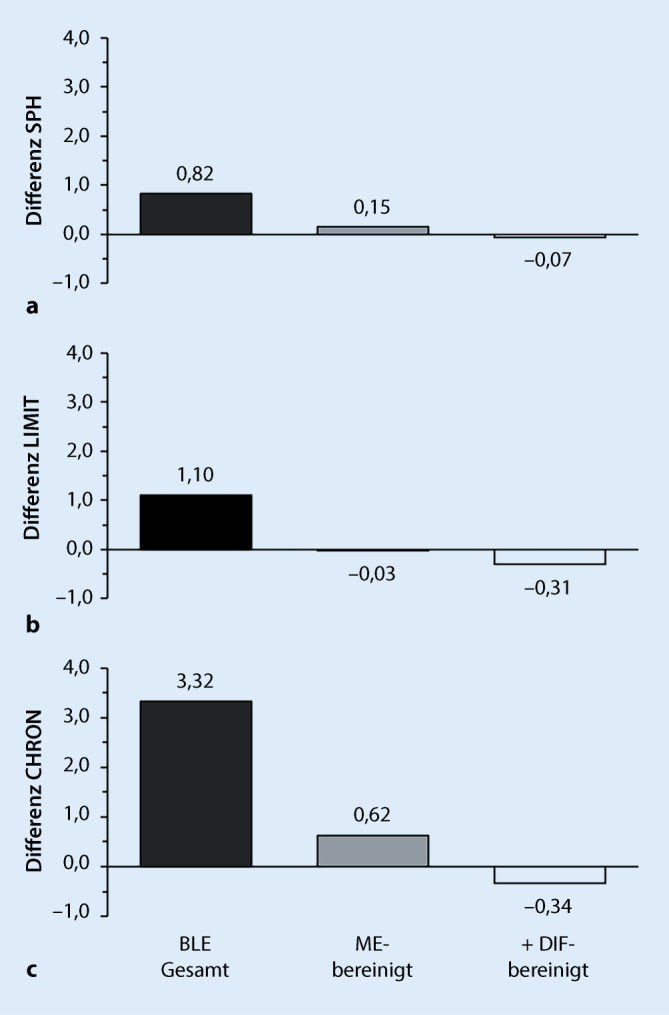

In Abb. 2 sind die Ergebnisse der 3 Analyseschritte noch einmal grafisch zusammengefasst. Dabei ist für jede Variante der beeinträchtigten Lebenserwartung im Alter 50 – Abb. 2a für SPH, Abb. 2b für LIMIT und Abb. 2c für CHRON – die Frauen-Männer-Differenz dargestellt: in den schwarzen Säulen für die gesamte Anzahl an beeinträchtigten Lebensjahren, in den grauen Säulen bereinigt um den Mortalitätseffekt und in den weißen Säulen mit zusätzlicher Bereinigung um den DIF-Effekt. Dabei ist zu erkennen, wie sich die ursprünglich höheren Werte für die Frauen bei allen 3 Varianten der beeinträchtigten Lebenserwartung schrittweise reduzieren und nach Bereinigung um ME und DIF-Effekte sogar zu geringeren Werten bei allen 3 Gesundheitsindikatoren wandeln. Dabei ist das Ausmaß der Differenz mit einer nun für Männer höheren Anzahl an beeinträchtigten Lebensjahren nach ME- und DIF-Bereinigung sogar bei den chronischen Krankheiten (CHRON) am größten, wo sich in den ursprünglichen BLE-Werten noch die ausgeprägteste Differenz zuungunsten der Frauen zeigte.

## Diskussion

Die Vorstellung eines widersprüchlichen Verhältnisses zwischen den Geschlechterdifferenzen in der Lebenserwartung mit mehr Lebensjahren für Frauen, was seit der Jahrtausendwende in allen Ländern der Erde zu beobachten ist [35], und in den ungesunden Lebensjahren, bei denen die Frauen ebenfalls höhere Werte als die Männer aufweisen, hat sich fest in der Sichtweise von Forschenden und der interessierten Allgemeinheit etabliert. Dass sich trotz der in der Einleitung erwähnten Zweifel das Bild eines generellen Geschlechterparadoxons so hartnäckig hält, ist wohl darauf zurückzuführen, dass trotz der zahlreichen Forschungen noch immer sehr wenig über die Gründe oder seine Mechanismen bekannt ist [36–38].

Mit dem vorliegenden Beitrag wurde versucht, zum Schließen dieser Forschungslücke beizutragen und eine Erklärung für die widersprüchlich wirkenden Geschlechterdifferenzen in gesamter und gesundheitlich beeinträchtigter Lebenserwartung zu liefern. Die präsentierten Ergebnisse der empirischen Analysen legen nahe, dass sich dieses Paradoxon bezüglich der betrachteten Varianten der beeinträchtigten Lebensjahre durch die 2 analysierten Erklärungsfaktoren – 1) den Mortalitätseffekt, der besagt, dass die höhere Lebenserwartung der Frauen zu einer höheren Anzahl an mit gesundheitlichen Beeinträchtigungen verbrachten Lebensjahren führt, und 2) die Geschlechterdifferenzen im Gesundheitsberichtsverhalten – nicht nur teilweise, sondern vollständig auflösen lässt. Während der Mortalitätseffekt als wesentliche Ursache für die höhere Anzahl an gelebten Jahren mit gesundheitlichen Beeinträchtigungen seitens der Frauen erst in jüngerer Zeit thematisiert wurde [20, 22, 23, 39], gehört die höhere Sensibilität der Frauen und ihre ausgeprägtere Offenheit, Gesundheitsprobleme zu berichten, schon länger zu den Erklärungsversuchen für das Geschlechterparadoxon (siehe z. B. [10, 18, 40, 41]). Mit den Ergebnissen zum Mortalitätseffekt bestätigt die Studie somit die Hypothese von Luy und Minagawa [20], wobei diese in dem vorliegenden Beitrag für 3 statt einem globalen Gesundheitsindikator und nicht indirekt durch Korrelation, sondern direkt durch Dekomposition getestet wurde. Der innovativste Beitrag des vorliegenden Artikels liegt jedoch in der Quantifizierung des so sogenannten Differential Item Functioning (DIF) mithilfe von Gesundheitsvignetten und seiner Berücksichtigung in Form der Schätzung einer DIF-bereinigten Geschlechterdifferenz in der beeinträchtigten Lebenserwartung.

Gerade bezüglich Letzterer ist es jedoch wichtig zu betonen, dass es sich bei der hierfür entwickelten und angewandten Methode nur um eine approximative Schätzung des DIF-Effekts handelt. Die Schätzung der DIF-Anpassungsfaktoren für die 3 Gesundheitsindikatoren des MEHM auf Basis der in den Vignetten beschriebenen Gesundheitsmerkmale erfordert eine Vielzahl von Annahmen, die zum Teil von nicht unerheblichen Unsicherheiten begleitet werden. Auch wird der Vignettenansatz generell als Instrument für die Standardisierung von heterogenem Berichtsverhalten in Surveys infrage gestellt, was in der Fachliteratur unter den Bezeichnungen „vignette equivalence“ (homogenes Verständnis bezüglich der Schwere von Gesundheitsproblemen unter den Befragten) und „response consistency“ (Verwendung einer einheitlichen Skala zur Bewertung des Gesundheitszustands sowohl der Vignettencharaktere als auch der Befragten selbst) diskutiert wird (Details zu all diesen Aspekten sind bei [28] zu finden). Die präsentierten Ergebnisse dürfen daher nicht als gesicherte Belege für das Vorliegen geschlechtsspezifischer Unterschiede im Berichtsverhalten oder als verbesserte Schätzung der beeinträchtigten Lebenserwartung verstanden werden. Ebenso ist zu berücksichtigen, dass in den in dieser Arbeit präsentierten Analysen angenommen wurde, dass es sich bei Frauen und Männern um homogene Bevölkerungsgruppen handelt. Obwohl diese Annahme den meisten bisherigen Forschungen zu geschlechtsspezifischen Unterschieden in Gesundheit und Sterblichkeit zugrunde liegt, ist ihre Richtigkeit zumindest zu bezweifeln. Forschungsansätze, die eine Heterogenität innerhalb der Geschlechtergruppen aufgreifen, sollten daher in zukünftigen Arbeiten aufgegriffen werden. Auch kann diese Studie keine für alle Bevölkerungen gültige Schlussfolgerung in Anspruch nehmen, dass sich die widersprüchlichen Geschlechterdifferenzen in Lebenserwartung und gesundheitlich beeinträchtigten Lebensjahren allein durch den Mortalitätseffekt und das unterschiedliche Berichtsverhalten von Frauen und Männern erklären lassen. Zumindest aber gilt dies für die deutsche Bevölkerung des Jahres 2012 und die Daten, mit denen die vorgestellten Analysen durchgeführt wurden. Studien mit anderen Datensätzen, für andere Kalenderjahre und andere Bevölkerungen sind erforderlich, um die Generalisierbarkeit der präsentierten Ergebnisse und Schlussfolgerungen zu verifizieren. Schließlich muss erwähnt werden, dass die vorliegende Studie auf den sehr breiten Gesundheitsindikatoren Allgemeingesundheit (SPH), gesundheitsbedingte Einschränkungen im Alltagsleben (LIMIT) und das Leben mit einer oder mehreren chronischen Krankheiten (CHRON) basiert und auch diesbezüglich nicht von einer Übertragbarkeit auf alle anderen Gesundheitsausprägungen ausgegangen werden kann. Dies gilt besonders für die vielen kleinen und spezifischen Erkrankungen und Gesundheitsprobleme, für die Geschlechterdifferenzen existieren und die sich nicht in den hier betrachteten breiten Gesundheitsmerkmalen wiederfinden.

## Fazit

Die Ursachen für die Geschlechterunterschiede in Lebenserwartung und gesundheitlich beeinträchtigten Lebensjahren sind sehr komplex und die diesbezüglichen Frauen-Männer-Differenzen sind nicht so klar gegensätzlich gerichtet, wie es der Begriff des Geschlechterparadoxons suggeriert. Das Ausmaß der höheren Anzahl an gesundheitlich beeinträchtigten Lebensjahren bei Frauen hängt entscheidend vom zugrunde liegenden Gesundheitsindikator ab. Diesbezüglich decken sich die hier präsentierten Ergebnisse mit jenen der Studie von Gorman und Ghazal Read [11] zur US-amerikanischen Bevölkerung, die ebenfalls ein unterschiedliches Ausmaß der gesundheitlichen Geschlechterdifferenzen mit ungünstigeren Werten für Frauen bei den von ihnen betrachteten Gesundheitsindikatoren festgestellt haben. Als Grund für diese Variabilität in Abhängigkeit von der Gesundheitsdimension wurde jüngst die Schwere der mit der jeweiligen Gesundheitsausprägung verbundenen Beeinträchtigungen vermutet, die sich letztlich im Sterberisiko widerspiegelt [22, 42]. Dies schließt den Kreis zu der wichtigen Erkenntnis dieses Artikels, dass das Ausmaß der höheren beeinträchtigten Lebenserwartung der Frauen zum größten Teil durch den Mortalitätseffekt erklärt wird, wie dies bereits von Luy und Minagawa [20, S. 17] mit den Worten: „women suffer from worse health than men do not in spite of living longer, but because they live longer“, beschrieben wurde.

## Footnotes

1 Daneben enthielt nur noch die zweite SHARE-Welle Gesundheitsvignetten, dies jedoch nur in selektierter und dadurch in für die hier durchgeführte Analyse nicht nutzbarer Form.

2 SHARE beinhaltet für alle Vignetten eine männliche und eine weibliche Variante der Vignettencharaktere, wobei diese zufällig in gleicher Häufigkeit den Survey-Teilnehmerinnen und -Teilnehmern vorgelegt wurden. Für die in dieser Arbeit durchgeführten Analysen wurden die weiblichen und männlichen Vignetten für dasselbe Gesundheitsproblem zusammengefasst. Eine Geschlechtertrennung erfolgte lediglich für die Survey-Teilnehmerinnen und -Teilnehmer.

3 Bei diesem als Standard etablierten Signifikanzniveau handelt es sich um einen willkürlichen Grenzwert, der nur sehr bedingt Rückschlüsse auf die Relevanz der Assoziation erlaubt. Deswegen haben Luy et al. [28] neben der einfachen logistischen Regression auch das Lasso-Verfahren („least absolute shrinkage and selection operator logistic regression“) verwendet, um der willkürlichen Festlegung eines Grenzwertes zu begegnen. Dabei stellte sich heraus, dass die beiden Methoden zu keinen Unterschieden in den Ergebnissen führen.
